# Palm Kernel Shell Activated Carbon as an Inorganic Framework for Shape-Stabilized Phase Change Material

**DOI:** 10.3390/nano8090689

**Published:** 2018-09-05

**Authors:** Ahmad Fariz Nicholas, Mohd Zobir Hussein, Zulkarnain Zainal, Tumirah Khadiran

**Affiliations:** 1 Materials Synthesis and Characterization Laboratory, Institute of Advanced Technology (ITMA), Universiti Putra Malaysia, 43400 Serdang, Selangor, Malaysia; ahmadfariznicholas@yahoo.com (A.F.N.); zulkar@upm.edu.my (Z.Z.); 2 Forest Product Division, Forest Research Institute Malaysia (FIRM), 52109 Kepong, Selangor, Malaysia; tumirah@frim.gov.com

**Keywords:** activated carbon, palm kernel shell, phase change material, thermal energy storage, paraffin

## Abstract

The preparation of activated carbon using palm kernel shells as the precursor (PKSAC) was successfully accomplished after the parametric optimization of the carbonization temperature, carbonization holding time, and the ratio of the activator (H_3_PO_4_) to the precursor. Optimization at 500 °C for 2 h of carbonization with 20% H_3_PO_4_ resulted in the highest surface area of the activated carbon (C20) of 1169 m^2^ g^−1^ and, with an average pore size of 27 Å. Subsequently, the preparation of shape-stabilized phase change material (SSPCM-C20) was done by the encapsulation of n-octadecane into the pores of the PKSAC, C20. The field emission scanning electron microscope images and the nitrogen gas adsorption-desorption isotherms show that n-octadecane was successfully encapsulated into the pores of C20. The resulting SSPCM-C20 nano-composite shows good thermal reliability which is chemically and thermally stable and can stand up to 500 melting and freezing cycles. This research work provided a new strategy for the preparation of SSPCM material for thermal energy storage application generated from oil palm waste.

## 1. Introduction

Malaysia, Indonesia, Thailand, African countries like Nigeria, Cameroon and several Southern provinces of China are among the top producers of palm oil in the world [1]. As the leading producer and supplier of the oil palm, Malaysia keeps on increasing the production of palm oil by developing oil palm plantations beginning with only 54,000 hectares in the early 1960s, expected to increase to 5.1 million hectares by 2020 [2]. The oil palm industries in Malaysia are producing about 90 × 10^6^ tons of lignocellulosic biomass each year, of which empty fruit bunch (EFB), oil palm trunk (OPT) and oil palm fronds (OPF) are about 40 × 10^6^ tons [3]. It is an opportunity for oil palm industries to use these wastes to turn them into valuable products instead of discarding them by open burning [4] which resulted in bad consequences for the environment. The increasing number of waste material from industrial development contributed to the deterioration of the earth through ozone depletion. Oil palm solid waste (OPSW) like palm kernel shell is a good candidate for the production of activated carbon as it contains a highly developed porosity and surface area because of its high carbon content and relatively low price [5].

Palm kernel shell is a sustainable source of materials included in the economic sector which is responsible for the breakdown of global greenhouse gas emissions and climate change. Many industries, academics, and governmental agencies are now focusing on the green chemistry and engineering technologies to minimize the negative impacts towards the environment [6]. The manufacturing processes are now restructured by the application of green solvents and reagents, energy conservations, waste minimization, and utilization of natural resources.

Consisting of about 87% to 97% of carbon and other elements such as hydrogen, oxygen, sulfur and nitrogen, activated carbon has a very high adsorption capability by having the highest volume of adsorbing porosity [7]. Activated carbon with highly developed porosity, has a large surface area, a high micropore volume (V_mic_), a favourable pore size distribution, and thermal stability [8]. The surface area of activated carbon is usually more than 1000 m^2^/g. Activated carbon has a random imperfect structure which consists of a broad range of pore sizes—micro, meso, and macro—that makes activated carbon different from graphite [7] or other carbon materials. The surface of the activated carbon can be used to accumulate contaminants as it contains the protonated (C–OH_2_^+^), neutral (COH), or ionized (CO^−^) groups [9].

Activated carbon can be prepared using three main methods: chemical, physical and physicochemical activations. Researchers used the chemical method at the beginning of the activated carbon production era, but recently the physical and physicochemical methods are more preferred for the production of activated carbon, especially from oil palm solid waste. Chemical activation is the most widely used for the production as it provides a superior quality, high surface area, high porosity, and higher carbon yield [10]. Activating agents such as KOH, ZnCl_2_, K_2_CO_3_, and H_3_PO_4_ are usually used as they help to develop the pore structure of the activated carbon [11]. The most popular chemical activating agents that were used for the activation of activated carbon are ZnCl_2_ and H_3_PO_4_. It inhibits the tar formation and widens the porous structure of the activated carbon by increasing the carbon yield. The chemical activation by KOH and K_2_CO_3_ show different mechanism as the reaction with carbon causes carbon gasification and the formation of hydrogen, which will not contribute to the increase in carbon yield [12].

Controlling the activation process by chemical treatment will enable one to enhance and provide higher specific surface properties to the resulting activated carbon [13]. In chemical activation, an acidic or basic solution is used as the activating agent to produce a higher surface area and porosity [8]. There are several activating agents which are usually used in treating the OPSW such as H_3_PO_4_, K_2_CO_3_, KOH, ZnCl_2_, and H_2_SO_4._ The synthesis method will determine the resulting physicochemical characteristics of the activated carbon as it depends on the activating agent, the amount of the precursor, the condition of the activation process, and the raw materials used [7]. Proper chemical management is needed to discharge the activating agent after sample treatment in order to prevent pollutions. There are several methods that can be adapted to dispose of this highly polluting effluent, such as the treatment system based on membrane technology [14], in situ solvent and reagent recycle by Nanofiltration [15], or by solvent recycle with imperfect membranes by the separation method [16].

Thermal energy storage is an energy storage device that functions to develop a new energy source which is very important to help in conserving energy which, in turn, reduces the negative impact towards the polluted environment [17]. Phase change material (PCM) is the medium for latent heat thermal energy storage in which the energy stored will be used based on energy supply and demand when there is a change of phase caused by the freezing or melting processes [18]. Solid-liquid PCM such as n-octadecane is very useful as it can store a large quantity of energy with small changes of volume. In addition, it also possesses desirable properties such as a high latent heat, chemical inertness, no phase segregation, and it is commercially available [19]. In the preparation of the SSPCM nano-composite, the PCM was encapsulated into the activated carbon pores or inorganic frameworks to prevent any leakage during the solid-liquid phase transition.

In this work, the preparation of activated carbon from palm kernel shell will be studied and the resulting activated carbon will be subsequently used as inorganic frameworks for the preparation of shape-stabilized phase change material (SSPCM) for the thermal energy storage (TES) application. Parametric optimization for the preparation of activated carbon was done in order to obtain the highest graphitic content with the highest surface area and porosity. Three different parameters, namely, activation temperature, activation holding time, and concentration of H_3_PO_4_ were optimized.

## 2. Materials and Methods

### 2.1. List of Materials

Palm kernel shell (PKS), deionized water, orthophosphoric acid (85%) (SystermChemAR, Shah Alam, Malaysia), ethyl alcohol (99.7%) (R&M Chemicals, Semenyih, Malaysia), and octadecane (99%) (Aldrich Chemistry, St. Louis, MO, USA) were used in this work.

### 2.2. Pre-Treatment of Palm Kernel Shell

The palm kernel shell (PKS) samples were collected from the Seri Ulu Langat Palm Oil Mill, Dengkil, Selangor, to be used as the precursor for the activated carbon production. The PKS was cleaned using water, followed by de-ionized water. The sample was crushed into powder using a stable arm grinder before it was treated with H_3_PO_4_. About 20 g of the precursor was weighted and treated with 100 mL of freshly prepared H_3_PO_4_ at various concentrations of H_3_PO_4_: 0%, 5%, 10%, 20%, 30%, and 40% (*v*/*v*) using 85% H_3_PO_4_. Treatment was done in a conical flask with the ratio of 1:5 of 20 g of PKS and 100 mL of freshly prepared H_3_PO_4_. After 24 h of treatment, the liquid was filtered off using a filter paper and the sample was dried in an oven at 70 °C for 24 h before it was used as the precursor for AC preparation.

### 2.3. Sample Activation

An electrical tubular furnace was used in this one step activation with a constant flow rate of nitrogen gas at 150 cm^3^ min^−1^. The sample was activated in three different conditions based on the parameters to be optimized and this will be described in the following section.

#### 2.3.1. Carbonization Temperature

The PKS samples of about 5 g were carbonized using a tubular furnace under a nitrogen gas environment. The sample treated with 20% H_3_PO_4_ was chosen to study the effect of the carbonization temperature on the physicochemical properties of the activated carbons. The sample was carbonized at different temperatures: 500 °C, 600 °C, 700 °C, 800 °C, and 900 °C for 2 h with a holding time at a 10 °C min^−1^ heating rate. The resulting PKSACs prepared were crushed using a mortar and pestle before they were cleaned. The cleaned samples were oven-dried at 110 °C for 24 h to remove any remaining internal moisture. The samples were weighted and recorded again before they were kept in vials and labelled as T500, T600, T700, T800, and T900 for the sample prepared at carbonization temperatures of 500 °C, 600 °C, 700 °C, 800 °C, and 900 °C, respectively, for further used and various analyses.

#### 2.3.2. Carbonization Holding Time

For this experiment, the sample that was treated with 20% of H_3_PO_4_ was subsequently chosen and carbonized at 500 °C at different holding times: 1 h, 2 h, 3 h, 4 h, and 5 h at a 10 °C min^−1^ heating rate. The as-produced activated carbons were crushed using a mortar and pestle before they were further cleaned. The cleaned samples were oven dried at 110 °C for 24 h to remove any internal moisture left during the preparation. The samples were then weighted and recorded again before they were kept into vials and labelled as H1, H2, H3, H4, and H5 for holding times of 1 h, 2 h, 3 h, 4 h, and 5 h, respectively, for further used and various analyses.

#### 2.3.3. Concentration of the Activating Agent, H_3_PO_4_

Each treated sample that was prepared at different concentrations of H_3_PO_4_ was carbonized at 500 °C for 2 h of a holding time and at a 10 °C min^−1^ heating rate. The resulting PKSACs were crushed using a mortar and pestle before it was cleaned. The cleaned samples were then oven-dried at 110 °C for 24 h to remove any remaining internal moisture. The samples were then weighted and recorded again before they were kept into vials and labelled as C0, C5, C10, C20, C30 and C40 for treatment with H_3_PO_4_ at 0%, 5%, 10%, 20%, 30%, and 40%, respectively for further used and various analyses.

### 2.4. Preparation of Shape-Stabilized Phase Change Materials

A shape-stabilized phase change material (SSPCM) nano-composite was prepared by a simple impregnation method. About 30 mL of absolute ethanol was used to dissolve melted n-octadecane by heating above the melting temperature of n-octadecane above 28–30 °C. The prepared PKSAC, C20 was added into the n-octadecane solution and the solution was then stirred at 600 rpm for 4 h. The mixture was oven-dried at 80 °C for 48 h or until all the excess ethanol was evaporated. The SSPCM-C20 prepared was stored in a sample bottle for further used and characterization.

### 2.5. Characterization Method

The chemical characterization of PKSAC was carried out using the Raman spectroscopic method with a WiTec Raman spectrometer (WiTec, Ulm, Germany) using a 514 nm laser. The intensity ratio between the D-line (~1350 cm^−1^) and the G-line (~1597 cm^−1^) of the Raman spectra was used to evaluate the graphitic character of the PKSAC. In this study, characterizations were done for all the PKSACs prepared from the three different parameters: carbonization temperature, carbonization holding time, and concentration of the activating agent.

The surface area and porosity of PKSAC and SSPCM nano-composites were determined using the BET nitrogen gas adsorption–desorption method at 77 K using a Micromeratics Tristar II plus (Micromeratics, Norcross, GA, USA). The method was also conducted to identify whether the PCM, n-octadecane, can be impregnated into the newly prepared PKSAC pores or only adsorbed on the surface. The samples were degassed at 290 °C for 9 h under vacuum before the measurements and the specific surface area and pore size distribution of the samples was determined using the Brunauer-Emmet-Teller (BET) and BJH (Barret-Joyner-Halenda) Equations, respectively.

The chemical properties of the PKSAC and SSPCM nano-composites were carried out using the fourier-transform infrared spectroscopy (FTIR) on a Perkin Elmer BX FTIR spectrophotometer (Waltham, MA, USA) with the KBr method at room temperature. The absorbance was recorded from 400–4000 cm^−1^.

The powder X-ray diffraction (PXRD) patterns of PKSAC, n-octadecane, and SSPCM were obtained using a Shimadzu XRD-6000 PXRD (Kyoto, Japan) at room temperature with a scanning range of 10–35° (2θ) and a scanning rate of 4° min^−1^ for all samples. 

TGA/DTG thermal analyses were obtained using a Q500 V20.13 Build 39 (TA Instruments, Lukens Drive, New Castle, DE, USA) to investigate the thermal stability of the PKSAC, n-octadecane and SSPCM. The experiment was conducted using 10 mg of samples and heated under the nitrogen atmosphere at a 5 °C min^−1^ heating rate from room temperature to 1000 °C.

The thermal storage properties, such as the melting temperature, freezing temperature and enthalpy (latent heat) of the pure n-octadecane and SSPCM nano-composite were measured by a differential scanning calorimeter (DSC), 822e, Mettler Toledo equipped with a refrigerated cooling system. About 6 ± 1 mg of sample was weighed into an aluminium pan. Under a constant flow of nitrogen atmosphere with the flow rate of 60 mL/min, the measurements were performed at −20 to 70 °C for the heating cycle stage and 70 to −20 °C for the cooling cycle stage. The encapsulation efficiency of n-octadecane in the nano-composite s was calculated based on the enthalpy of pure n-octadecane using the following equation:

(1) $$\;\mathrm{PCM}\;\mathrm{content}\;\mathrm{in}\;\mathrm{nano}-\mathrm{composite}\;\left(\mathrm{wt}\;\%\right)\;=\;\left(\Delta H_{m}/\Delta H_{\mathrm{PCM}}\right)\;\times \;100\;$$

where ΔH_m_ is the enthalpy of melting of the SSPCM nano-composite (Jg^−1^) and ∆H_PCM_ is the enthalpy of melting for the pure n-octadecane (Jg^−1^).

The external surface morphology and microstructure of the PKS, PKSAC and SSPCM nano-composites were obtained using a Nova Nanosem 230 field emission scanning electron microscope (FESEM). The dried samples were dispersed on a conductive carbon adhesive tape surface that was attached to a FESEM stub and then gold-coated prior to the analysis.

To measure the chemical and thermal stability of the SSPCM nano-composite s to be used as a TES material, a thermal cycling test was conducted. This test is very important in determining a material’s service lifetime. The test was performed at a relative humidity of 70% which was maintained constant using a climatic chamber (Weiss Umwelt Technik, UK) equipped with a K-type thermocouple in the middle [20]. About 5 g of the dried SSPCM nano-composite was placed in contact with the thermocouple in a chamber. The accuracy of the temperature measurement was 0.1 °C. The nano-composite was subjected to 1000 thermal cycles at temperatures below and above the melting point of n-octadecane. Sampling was performed at 100, 300, 500, 700, and 1000 cycles. Changes in the samples’ thermal properties and chemical stability after the thermal cycling test were determined using the DSC and FTIR technique, respectively.

The leakage study was performed by keeping the SSPCM nano-composite inside an oven at 80 ± 5 °C for 3 days (72 h). Before that, about 1 g of the nano-composite was weighed on a filter paper and exposed to 30 °C for 8 h to study the ability of the C20 to hold n-octadecane during the melting phase. Then the samples were directly placed into an oven at 80 ± 5 °C for 3 days. The weight and the latent heat of the sample after the exposure period were recorded.

## 3. Results and Discussion

### 3.1. Raman Spectroscopy

The PKSAC samples were analyzed using the Raman spectroscopy to identify the graphitic character of the samples. The Raman spectra of all the PKSAC samples show two sharp peaks; a D band at around 1350 cm^−1^ and a G band at around 1597 cm^−1^ (Refer Figure S1: Raman spectra of PKSAC). The G band indicates the presence of a sp^2^ carbon network graphite-like structure which composed of either C=C chains or aromatic ring structures. The D band in the Raman spectrum is due to the disordered graphite structure which refers to defects of the graphitic characteristics that also exists in all the PKSAC samples.

**Figure 1:**
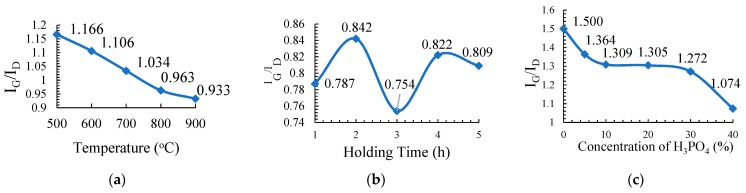
The plot of the I_G_/I_D_ against (**a**) carbonization temperature, (**b**) holding time, and (**c**) concentration of H_3_PO_4_ of the activated carbons.

Figure 1a presents the plot of I_G_/I_D_ versus temperature (°C) which shows that the graphitic character in the resulting PKSAC sample decreased almost linearly with the increased of the activation temperature. The highest graphitic content for the carbonization temperature is 500 °C with an I_G_/I_D_ value of 1.166 followed by 600 °C (1.106), 700 °C (1.034), 800 °C (0.963) and the lowest temperature of 900 °C (0.933). The graphitic value was found to decrease as the carbonization temperature increased and this is due to the reduction of amorphous carbon and the increase of the disorder aromatic carbon during the carbonization process [21].

Figure 1b presents the plot of I_G_/I_D_ against the holding time (h), which shows that no specific trend was observed between the graphitic characters in the PKSAC samples to the holding time. The highest value was obtained at 0.842 for the H2 sample followed by H4 (0.822), H5 (0.809), H1 (0.787), and H3 (0.754), the lowest. From the result, it is clear that the best holding time for the preparation of PKSAC with a high graphitic character is 2 h.

Figure 1c shows a plot of the I_G_/I_D_ value of PKSAC against a concentration of H_3_PO_4_ (% *w*/*w*) used for the activating agent for the PKSAC, indicating different graphitic contents of different samples of PKSAC. The highest graphitic character was observed for the sample prepared without any H_3_PO_4_ treatment, C0 (untreated sample) with a value of 1.5, followed by C5 (1.364), C10 (1.309), C20 (1.305), C30 (1.272), and C40 with the value of 1.074. Generally, it shows that the graphitic content of the PKSAC is indirectly proportional to the concentration of H_3_PO_4_. The higher the concentration, the lower the graphitic content in the resulting PKSAC and the weaker the physical structure of the sample.

### 3.2. Surface Area and Porosity

#### 3.2.1. Activated Carbon Framework

**Figure 2:**
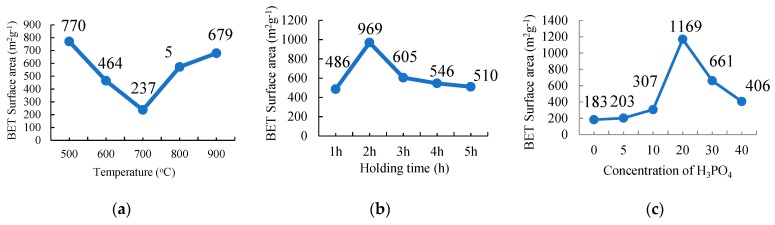
The plot of the Brunauer-Emmett-Teller surface area against (**a**) the carbonization temperature,(**b**) holding time, and (**c**) the amount of H_3_PO_4_ used as the chemical activator for the preparation of Palm kernel shell activated carbon.

The nitrogen adsorption-desorption isotherms of the three different parameters—carbonization temperature, holding time, and concentration of the activator—are shown in Figure 2a–c, respectively (Refer Figure S2: Adsorption-desorption isotherms). This study was carried out in order to get the highest surface area of the activated carbon for each of the parameters studied and finally identify the optimum temperature, activation holding time, and the concentration of H_3_PO_4_ treatment, in the preparation of the highest specific surface area of the activated carbon. All the samples are dominated by the Type 1 isotherm (according to the IUPAC classification) for all the parameters used in this study. The isotherm indicates that the AC is dominated by microporous-rich texture mixed with some of the mesopores with the highest BET specific surface area of 1168 m^2^ g^−1^ for the sample treated with 20% H_3_PO_4_ (C20).

Figure 2a shows the BET surface area of AC that was activated at 500 °C, which shows the highest value. The surface area of the ACs decreased from 770 m^2^ g^−1^ to 464 m^2^ g^−1^ and 237 m^2^ g^−1^ when it was carbonized at 600 and 700 °C. At 800 and 900 °C, the surface area increased to 572 m^2^ g^−1^ and 679 m^2^ g^−1^, respectively. This shows that 500 °C was the best temperature to prepare the highest surface area of the AC. This relatively low temperature of carbonization is also good to reduce the use of energy which will reduce the cost of manufacturing.

Similar to the carbonization holding time as shown in Figure 2b, a carbonization holding time less or more than 2 h results in a lower BET surface area value. It shows that a 1 h holding time is not enough time to manufacture more pores in the AC and when the samples were treated for more than 2 h, the BET surface area and porosity value reduced with the increasing holding time. The sample was overtreated, which destroyed the pore formation of the AC. The overtreated samples extend the size of the pores from micro- to meso- and macro-pores sizes, which reduced the surface area and porosity of the AC. In other words, the porous structure development does not reach its optimum treatment when the H_3_PO_4_ concentration and activation holding time are lower or higher than the values of 20% H_3_PO_4_ and a 2 h holding time.

The same result is shown with the concentration of the H_3_PO_4_ parameter (Figure 2c) in which the BET surface area of the AC treated with 20% H_3_PO_4_ resulted in the highest surface area and this was subsequently used as the frameworks for the encapsulation of the PCM, n-octadecane, for the formation of a shape-stabilised PCM (SSPCM).

Based on the parametric optimization for the preparation of PKSAC to be used as a framework for PCM, it was found that activation at 500 °C, with a 2 h activation holding time with H_3_PO_4_ of 20% treatment resulted in the highest surface area and welldeveloped porosity.

#### 3.2.2. Shape-Stabilized Phase Change Material

**Figure 3:**
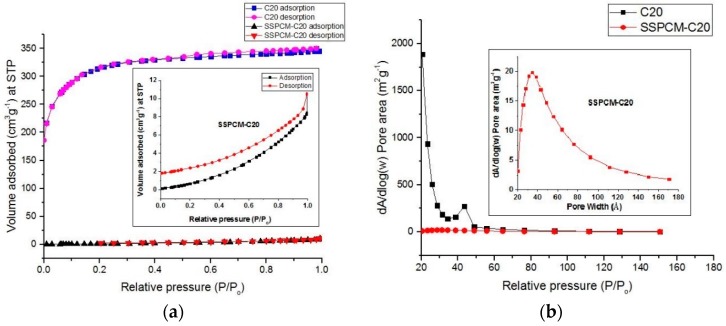
The (**a**) adsorption-desorption isotherm and (**b**) pore distribution of the activated carbon (C20) and its Shape-stabilized phase change material (SSPCM-C20). The insets show the expanded y-axis.

The preparation of SSPCM-C20 was done by the impregnation of n-octadecane into the pores of activated carbon, C20. The BET surface area and porosity analysis for SSPCM-C20 was conducted to identify the surface properties changes before and after the encapsulation process. The SSPCM-C20 nano-composite was degassed at 290 °C for 9 h under a vacuum before the specific surface area and pore size distribution by using the Brunauer-Emmet-Teller (BET) and BJH (Barret-Joyner-Halenda) equations were measured. The result shows that the adsorption-desorption isotherms transformed from the Type I in the AC-20 to the Type III in the SSPCM-C20 as shown in Figure 3a, indicating that the nano-composite is of a nonporous material. This material is parallel with almost no pore size distribution that can be clearly observed for SSPCM-C20 compared to C20 as shown in Figure 3b. The BET surface area, total pore volume, and pore diameter of the former are 2 m^2^ g^−1^, 0.012 cm^3^ g^−1^, and 51 Å, respectively.

This result indicates that there are huge physical changes of the C20 (as the host) before the encapsulation of the PCM for the formation of the SSPCM-C20, after the PCM (as the guest) was encapsulated into the pores of AC. The encapsulation caused a reduction of the BET surface area and porosity, as well as the transformation from a microporous material to a nonporous material, as indicated by the adsorption-desorption isotherm from Type I to Type III. These changes happened because the pores of C20 were fully occupied by the n-octadecane after the encapsulation process. This result clearly proves that the SSPCM-C20 nano-composite was successfully fabricated by the impregnation of n-octadecane into the pores of C20 frameworks.

The size of a single n-octadecane molecule was estimated using the Pymol software. The 3D molecular structure of n-octadecane had a maximum energy of 44.4874 KJ mol^−1^ with a size of (20.7 × 1.8 × 1.8) Å. This size proves that n-octadecane can be impregnated into the pores of the activated carbon that dominate with a 12.85 Å pore diameter.

### 3.3. Fourier-Transform Infrared Spectroscopy

**Figure 4:**
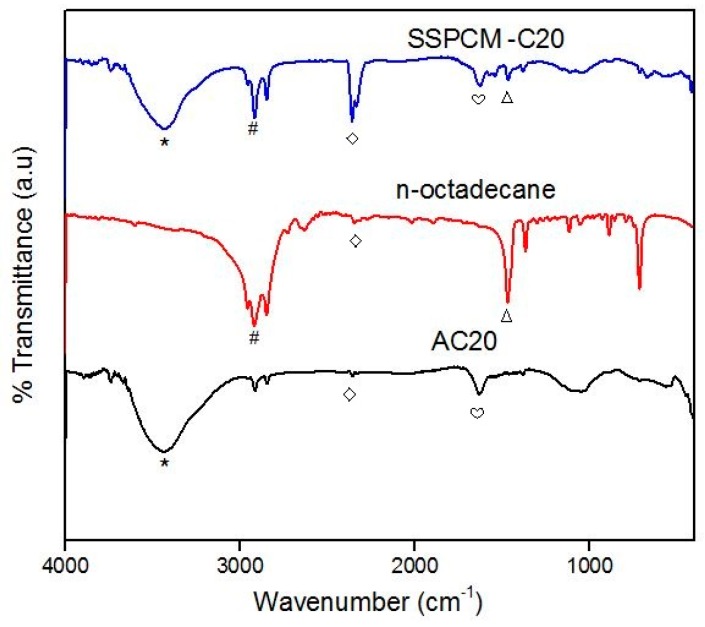
The FTIR spectra of C20, n-octadecane, and SSPCM-C20 (the symbols on the FTIR spectra are assigned to the FTIR adsorption peak; (⋆) 3430 cm^−1^, (**#**) 2919 cm^−1^, (◇) 2360 cm^−1^, (♡) 1635 cm^−1^, (Δ) 1470 cm^−1^.

The chemical characterization of the C20, n-octadecane, and SSPCM-C20 was carried out using the FTIR spectroscopy as shown in Figure 4. The main absorption band of C20 is observed in the region of 3650–2500 cm^−1^ due to the O–H stretching presumably due to H_3_PO_4_ treatment used and adsorbed moisture. The bands at 1635 and 1537 cm^−1^ correspond to the C=C stretching of the aromatic ring carbon of the polycyclic aromatic hydrocarbons that forms the basic structural unit of activated carbons. The absorption bands at 1463 cm^−1^ and 1385 cm^−1^ are due to the CH_2_ bending vibrations.

The FTIR spectra of n-octadecane exhibit C–H stretching in the region of 2975–2850 cm^−1^ corresponding to the main absorption band for the n-octadecane. The absorption band at 1470 cm^–1^ is due to the CH_2_ bending vibration in n-octadecane. The FTIR spectrum of the SSPCM-C20 nano-composite shows the combination of O–H and C–H stretching that corresponds to the absorption band in C20 and the n-octadecane. Based on the FTIR spectra, no chemical interaction between the n-octadecane and C20 can be observed. The possible interaction is due to capillary and surface tension forces between the AC and the paraffin, where the forces prevent the liquid leakage of the melted n-octadecane.

### 3.4. X-ray Diffraction

**Figure 5:**
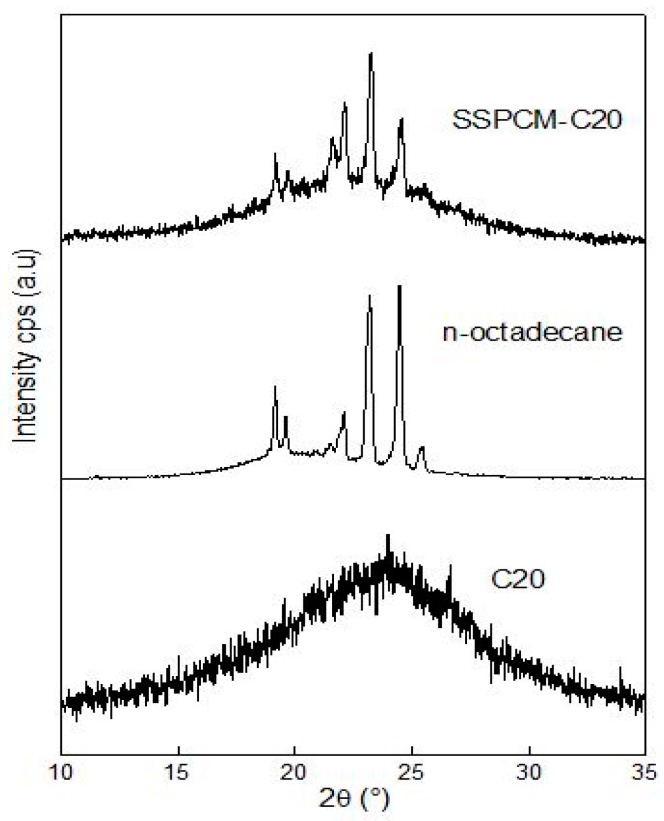
The X-Ray Diffraction patterns of C20, n-octadecane, and SSPCM-C20.

The XRD patterns of C20, n-octadecane, and the SSPCM-C20 nano-composite are displayed in Figure 5. The C20 was analyzed at room temperature while the n-octadecane and SSPCM-C20 were analyzed at 27 ± 5 °C in order to maintain the solid phase of PCM while we acquired the data. The result shows that there were no XRD peaks for the C20, as the material used in this study is of an amorphous type. In contrast, an XRD pattern for the SSPCM-C20 nano-composite is observed at 2θ = 19°, 23°, and 25°, which belongs to n-octadecane due to its crystallization in the solid phase condition. It can be seen that the XRD pattern of the SSPCM-C20 resembles that of the C20 and the n-octadecane. This infers that the n-octadecane was successfully impregnated into the pores of C20.

### 3.5. Thermal Properties

**Figure 6:**
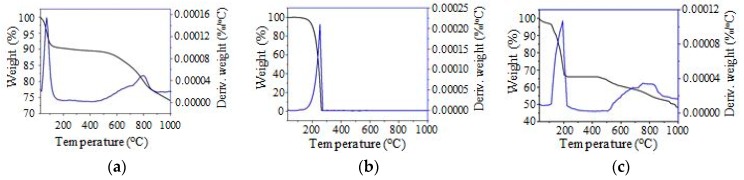
The Thermogravimetric analysis thermograms of (**a**) C20, (**b**) n-octadecane and (**c**) SSPCM-C20.

The TGA/DTG analysis was used to study the thermal stability of the SSPCM-C20. For the TES application, the thermal stability of the material is very important. Figure 6a–c show the TGA/DTG thermograms of C20, n-octadecane, and SSPCM-C20. C20 shows no thermal decomposition of the sample below 512 °C, however, a decomposition was observed from this temperature up to 861 °C due to further pyrolysis. This is because the carbonization of C20 was done at only 500 °C. This result indicates that the AC produced is thermally stable up to 513 °C. The C20 is thermally stable until 500 °C and able to be used as a supporting material for PCM. In contrast, the n-octadecane shows a weight loss between 94.00–253.7 °C, which is due to the evaporation of n-octadecane. The SSPCM-C20 nano-composite exhibits a similar thermal stability to that of C20. In general, the weight loss of the SSPCM-C20 nano-composite agrees well with the one obtained from the DSC result with an encapsulation efficiency of 32%.

Table 1 shows the percentage weight loss of C20, n-octadecane, and SSPCM-C20 upon being subjected to thermal treatment from room temperature to 1000 °C. The result shows that C20 exhibits a 25.08% weight loss; 8.49% from the evaporation of water and 16.59% from the decomposition of C20 at temperatures higher than 500 °C. It was found that 100% of pure n-octadecane was evaporated, while SSPCM-C20 shows a total weight loss of 51.16%, with 3.22% due to the evaporation of moisture, 31.96% due to the evaporation of n-octadecane, and 15.98% due to the decomposition of C20 at temperatures higher than 518 °C in the SSPCM-C20.

### 3.6. Differential Scanning Calorimetry

**Figure 7:**
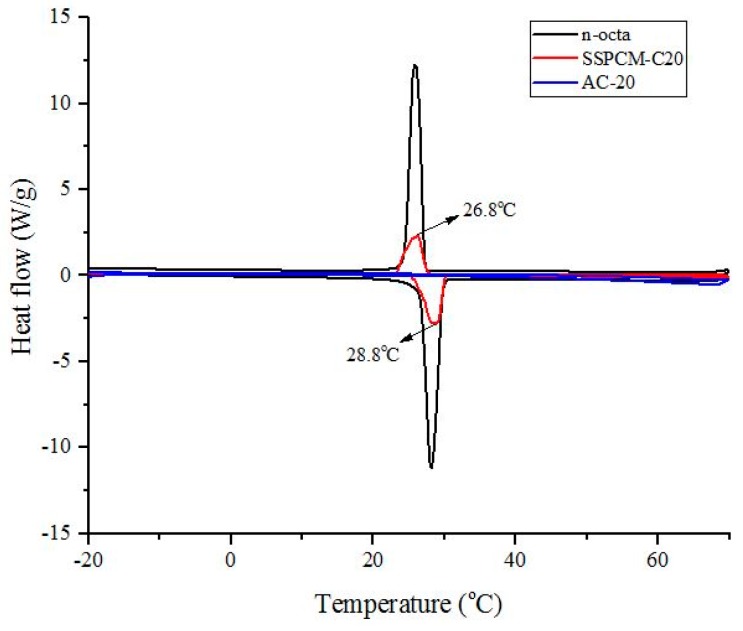
The Differential scanning calorimeter thermograms of C20, n-octadecane and SSPCM-C20.

The melting and freezing activity of the PCM was characterized using the differential scanning calorimeter (DSC) method. Figure 7 shows the DSC curves of the C20, n-octadecane, and SSPCM-C20. The DSC thermogram shows no activity of melting and freezing for C20 due to the absence of n-octadecane. In contrast, the DSC thermograms for n-octadecane and SSPCM-C20 shows peaks of solid-liquid melting and liquid-solid freezing which indicate that heat was absorbed and released for n-octadecane and SSPCM-C20. The pure n-octadecane shows sharper peaks compared to SSPCM-C20. This is because the AC that acted as a framework for the n-octadecane in SSPCM-C20 exhibits a higher adsorption capability compared to n-octadecane, which increases the capillary effect and surface tension forces between the n-octadecane and the porous network of the AC and hinders the molecular motion of n-octadecane during the melting and freezing processes.

The supercooling effect of the nano-composite was calculated by the difference between the melting point and the freezing temperature of the sample. The SSPCM nano-composite shows the melting and freezing temperatures of 28.8 °C and 26.8 °C, respectively, corresponding to the latent heat values of −87.42 Jg^−1^ and 84.31 Jg^−1^, respectively, indicating a very low supercooling effect. It also shows that pure n-octadecane and SSPCM-C20 have similar thermal properties, which indicates that there is no chemical reaction between them. This finding infers that n-octadecane was successfully impregnated into the pores of C20.

### 3.7. Surface Morphology

**Figure 8:**
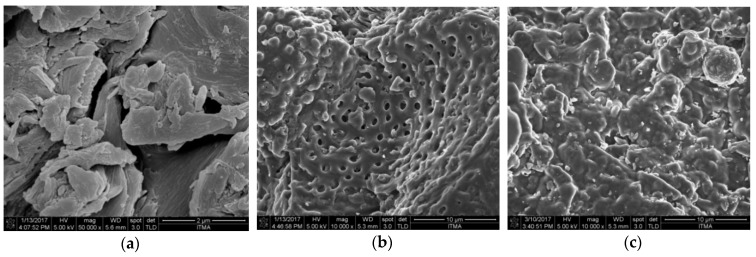
The Field Emission Scanning Electron Microscopy surface morphology images of (**a**) PKS (50000× magnification), (**b**) C20 and (**c**) SSPCM-C20 (viewed under 10,000× magnification).

Figure 8 shows the FESEM images of the PKS, C20, and SSPCM-C20 which show different morphologies of the samples. Figure 8a shows the morphology of the PKS, which was used as the precursor for the preparation of activated carbon. The resulting activated carbon prepared from PKS, activated using H_3_PO_4_, and carbonized at 500 for 2 h shows a well-developed porous structure with quite a similar shape and size with various pore sizes as shown in Figure 8b. As a result of the encapsulation of n-octadecane into the pores of activated carbon, n-octadecane was absorbed into the porous networks of C20 and completely dispersed and occupied homogeneously into the pores, resulting in the absence of pores.

The n-octadecane was uniformly distributed into the pores of AC due to the capillary effect and surface tension forces between n-octadecane and the pores of AC. In addition, the surface adsorption of n-octadecane on C20 cannot be ruled out. The porous structure of the C20 provides a mechanical strength which prevented the escape of the melted n-octadecane.

### 3.8. Leakage Study

The purpose of this test is to see the ability of the C20 to hold n-octadecane at temperatures over the melting temperature of n-octadecane. The filter paper shows no leakage, i.e., no oily marks on the paper and the sample is still in a dry condition after being exposed to 30 °C for 8 h. Table 2 summarizes the weight and latent heat of the SSPCM-C20 nano-composites before and after the leakage study. The weight after it was exposed to 80 °C for 3 days shows a reduction of 0.0409 g from 1 g of SSPCM-C20 used in this study. This insignificant difference of weight indicates that no leakage happened during the study. The latent heat of SSPCM-C20 before and after the leakage study also shows no significant difference. This is because the wall of the pores and n-octadecane provide strong adhesive forces that are built from a strong covalent bond that prevents any seepage of liquefied n-octadecane. Table 2 shows that the loading of n-octadecane is 34%, which is equivalent to the PCM that managed to be loaded into the pores of the activated carbon. This loading percentage of n-octadecane is similar to the weight loss percentage of n-octadecane in the TGA analysis. This result reconfirmed that the n-octadecane successfully penetrated and was encapsulated into the pores structure of PKSAC with a 34% loading value.

### 3.9. Thermal Cycling Test

The thermal cycling test was performed to investigate the physical and chemical stability of the SSPCM-C20 nano-composite when it was exposed to temperature changes. The repeated cycles of the temperature change the thermal behaviour of the material. Changes in the thermal behaviour of the nano-composite will affect its application as a TES. The number of cycles that the sample was able to withstand imitates the life-spend of the materials. For example, if there are no changes in the chemical or thermal properties of the nano-composite after 1000 cycles, the composite can be used in the building material to reduce and maintain the internal temperature for a minimum of 3 years. The higher cycle the SSPCM-C20 nano-composite can withstand, the better its application as a TES for building applications.

**Figure 9:**
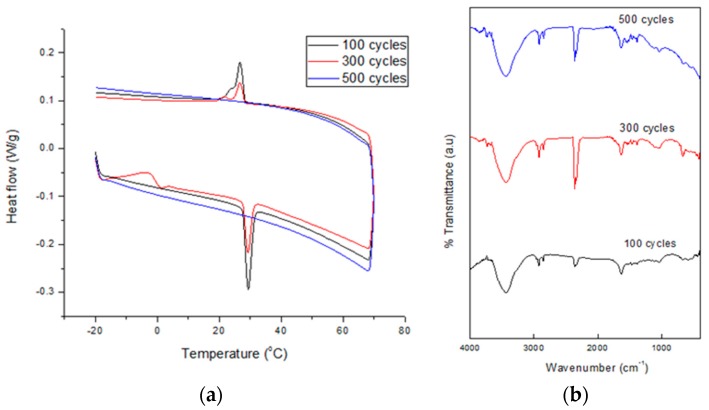
The (**a**) DSC thermograms and (**b**) FTIR spectra of SSPCM-C20 after 500 cycles of thermal cycling test.

Figure 9 shows the DSC thermograms (a) and FTIR spectra (b) of SSPCM-C20 after 100–500 cycles. The DSC thermograms show peaks of solid-liquid melting and liquid-solid freezing for 100–500 cycles. After 300 cycles, the melting and freezing peaks of the nano-composite were reduced, compared to 100 cycles. No melting and freezing activity for SSPCM-C20 was observed after 500 cycles. The peak keeps reducing until it disappeared at 500 cycles, which indicated that there are changes in the thermal properties of the SSPCM-C20 nano-composite. The FTIR spectra of the SSPCM-C20 nano-composite after 100, 300, and 500 cycles show similar FTIR features, indicating a similar chemical structure and that no chemical reaction between the C-20 and n-octadecane can be clearly observed.

## 4. Conclusions

The activated carbon prepared using PKS (PKSAC) as the precursor shows a good graphitic characteristic at the following optimum conditions: 20% H_3_PO_4_ (*w*/*w*) at 2 h holding time and 500 °C carbonization temperature. The nitrogen adsorption-desorption isotherm shows that the resulting PKSAC is micropore-rich as indicated by the Type I adsorption-desorption isotherm with the highest BET surface area of 1169 m^2^ g^−1^ and I_G_/I_D_ ratio of 1.305. When the resulting PKSAC was used as the frameworks for the encapsulation of the PCM material, n-octadecane, for the formation of a shape-stabilised PCM, the nitrogen adsorption-desorption isotherm changed to a non-porous material of Type III with the BET surface area reduced to only 2 m^2^ g^−1^. The change of the type of isotherm and the reduction of the BET surface area proves that the pores of the AC were fully occupied by the n-octadecane after the impregnation processes. The FESEM studies also show that the pores of the activated carbon were fully occupied by the PCM materials.

The interaction between the wall of the pores and the n-octadecane due to the strong covalent bond provides strong adhesive forces that prevent any seepage of the liquefied n-octadecane. The SSPCM-C20 is chemically and thermally stable, capable of standing up to 500 cycles. This result shows that the SSPCM-C20 nano-composite is thermally and chemically reliable to be used as a TES application. Due to the fact that the PKS was obtained from bio-wastes of the palm oil industry, the reproducibility is expected to be low. It is difficult to repeat and have exactly the same result for the experiment due to several aspects. The raw material used, processing methods and productions conditions will directly affect the physicochemical properties of the AC [22]. However, this experiment could be improved using other raw materials because the properties of the resulting AC produced are very much dependent on the precursor used. In addition, the further optimization on the preparation of AC framework can be accomplished using other precursors, activating agents, and different carbonization temperatures and holding times, so that a higher loading percentage of PCM and better thermal energy storage materials can be obtained.

## Figures and Tables

**Table 1:** The thermal properties of n-octadecane, C20, and SSPCM-C20 derived from Thermogravimetric analysis thermograms.

Sample Name	T_1_–T_2_ (^o^C)	T_m_ (°C)	Δ_m_ (mg)	Weight Loss (%)	Total Weight Loss (%)
C20	34–109	75	0.89	8.49	25.08
	513–861	796	1.74	16.59	
n-octadecane	94–273	261	10.69	100.00	100.00
SSPCM-C20	47–87	58	0.34	3.22	51.16
	115–263	193	3.38	31.96	
	518–853	761	1.69	15.98	

**Table 2:** The weight and latent heat of the SSPCM-C20 nano-composites before and after the leakage study.

Sample	BET Surface Area (m^2^ g^−1^)	Pore Volume (cm^3^ g^−1^)	Pore Diameter (Å)	PCM Loading (%)	Latent Heat Before (J g^−1^)		Latent Heat After (J g^−1^)	
					∆H_m_^a^ (J g^−1^)	∆H_c_^b^ (J g^−1^)	∆H_m_^c^ (J g^−1^)	∆H_c_^d^ (J g^−1^)
SSPCM-20	2	0.012	51	33.62	−87.42	84.31	−85.31	84.13

^a^ enthalpy on the DSC melting curve before the leaching test; ^b^ enthalpy on the DSC freezing curve before the leaching test; ^c^ enthalpy on the DSC melting curve after the leaching test; and ^d^ enthalpy on the DSC freezing curve after the leaching test.

## Supplementary Materials

The following are available online at http://www.mdpi.com/2079-4991/8/9/689/s1, Figure S1: Raman spectra of PKSAC, Figure S2: Adsorption-desorption isotherms.
