# Supplementary material for: Palm Kernel Shell Activated Carbon as an Inorganic Framework for Shape-Stabilized Phase Change Material
Source: Nanomaterials (Basel). 2018 Sep 5;8(9):689. doi: 10.3390/nano8090689 (PMC6165392; doi:10.3390/nano8090689)
Supplement: Supplementary file 1 [file nanomaterials-08-00689-s001.pdf]

Supporting Information

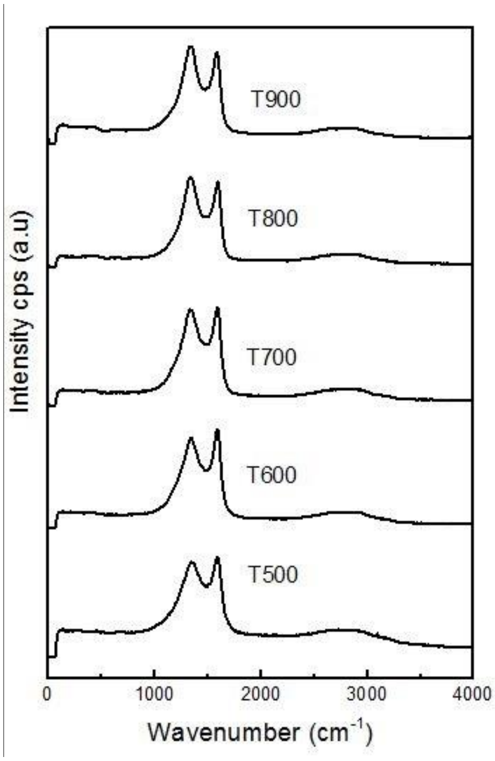

(a)

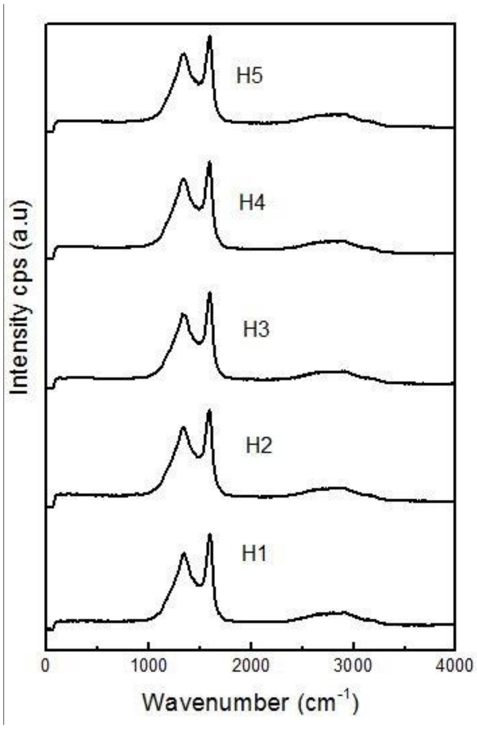

(b)

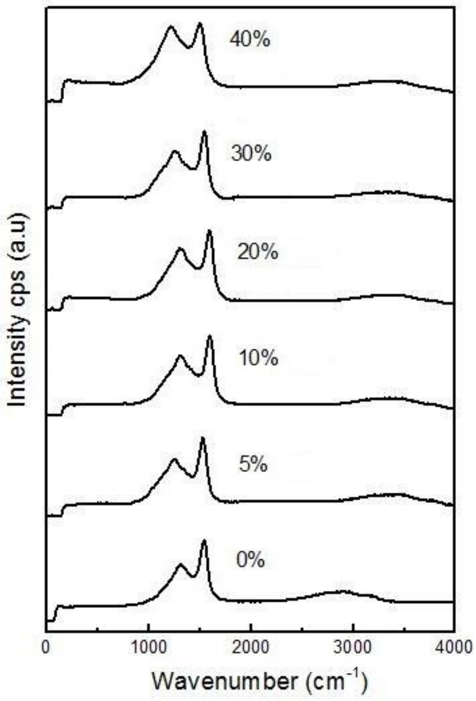

(c)

Fig.1 Raman spectra of PKSAC prepared using three different parameters (a) carbonization temperature, (b) carbonization holding time and (c) concentration of H_3PO_4.

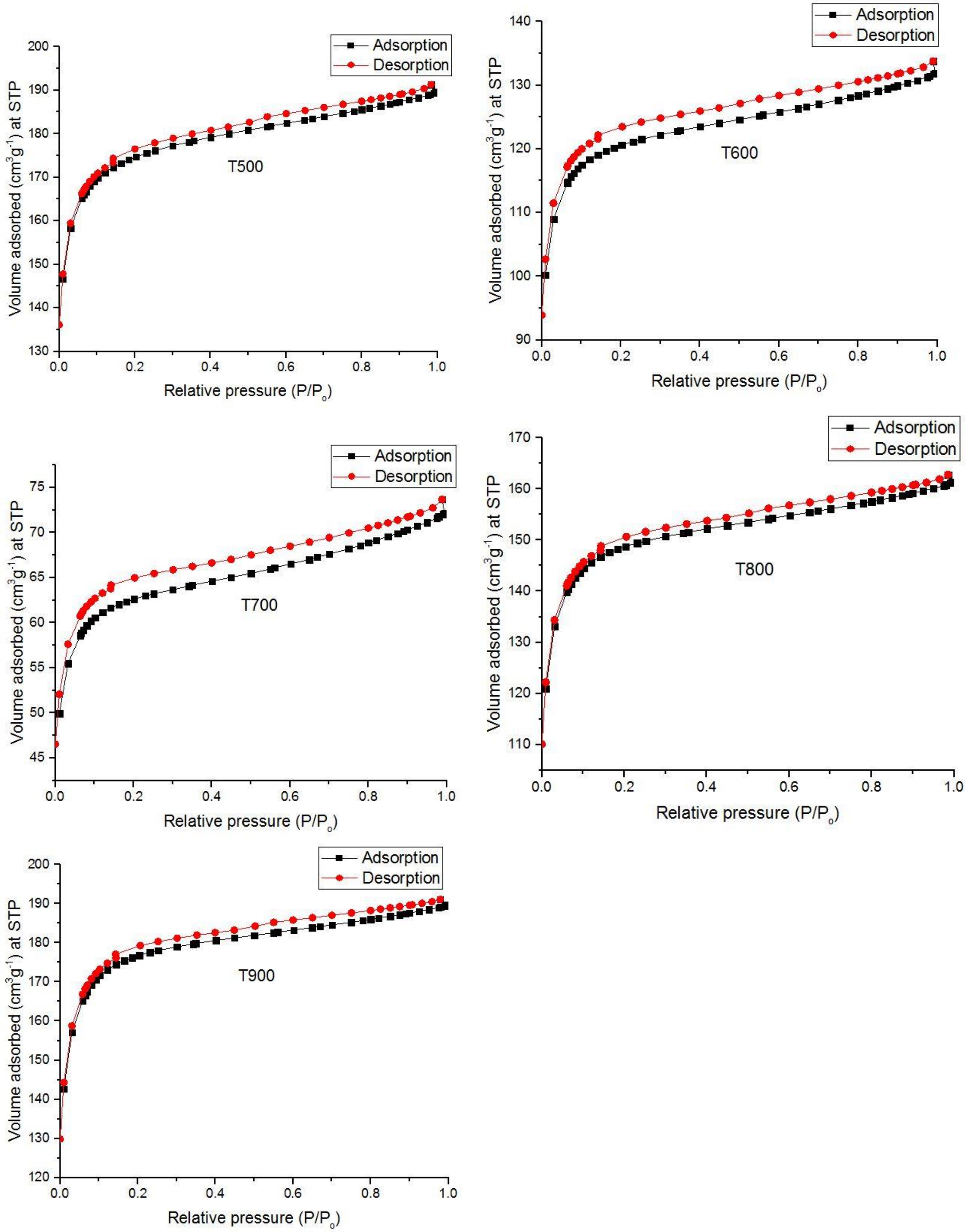

Fig.2a Adsorption-desorption isotherms of activated carbons prepared at various activation temperatures (^{o}C) as indicated by the last 3 digits of the sample's name.

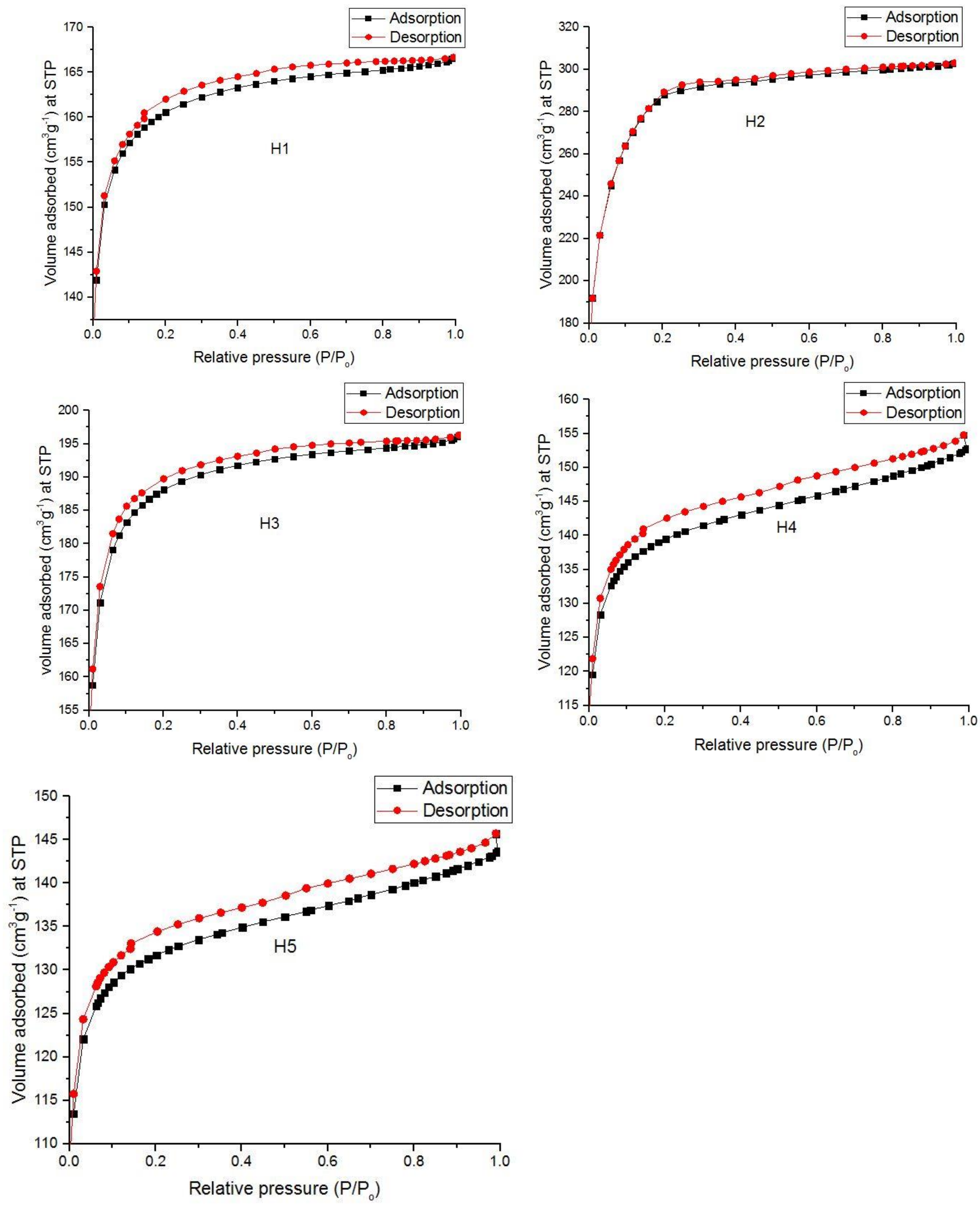

Fig.2b Adsorption-desorption isotherms of activated carbon prepared at various carbonisation holding times (h) as indicated by the last digit of the sample's name.

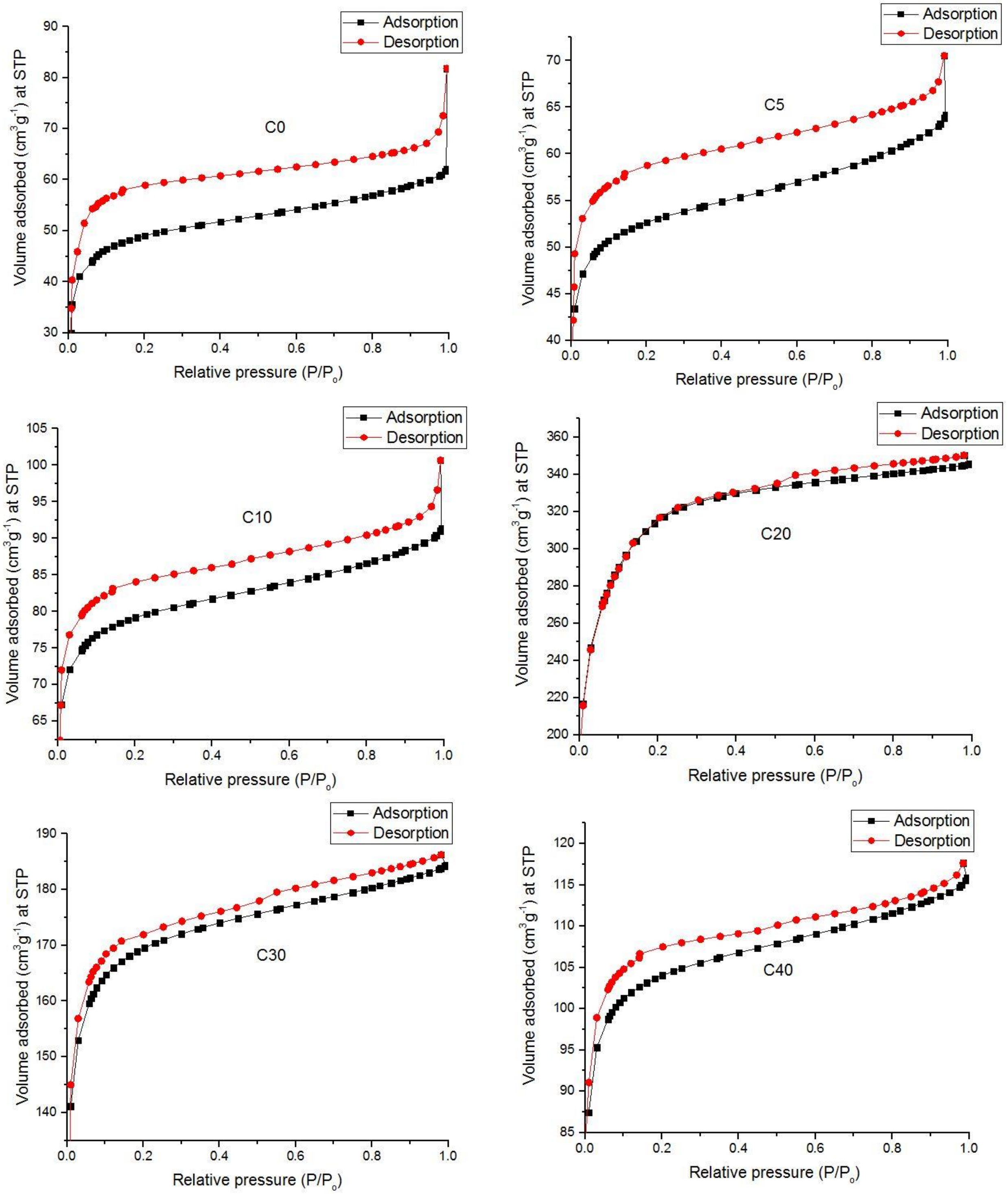

Fig.2c Adsorption-desoprtion isotherm of activated carbons prepared using various concentrations of H_3PO_4 (%) as indicated by the last 2 digits of the sample's name.
